# Using an ultra-compact optical system to improve lateral flow immunoassay results quantitatively

**DOI:** 10.1016/j.heliyon.2022.e12116

**Published:** 2022-12-07

**Authors:** Wei-Huai Chiu, Wei-Yi Kong, Yuan-Hui Chueh, Jyun-Wei Wen, Ciao-Ming Tsai, Chitsung Hong, Pang-Yen Chen, Cheng-Hao Ko

**Affiliations:** a Graduate Institute of Automation and Control, National Taiwan University of Science and Technology, Taipei, Taiwan; b Power Mechanical Engineering, National Tsing Hua University, Hsinchu, Taiwan; c SpectroChip Inc., Hsinchu, Taiwan; d Department of Emergency Medicine, Mackay Memorial Hospital, Taipei, Taiwan; e Institute of Public Health, School of Medicine, National Yang Ming Chiao Tung University, Taipei, Taiwan; f Department of Nursing, Yuanpei University of Medical Technology, Hsinchu, Taiwan

**Keywords:** Micro-spectrometer, MEMS, Rapid diagnosis, LFIA, POCT

## Abstract

The lateral flow immunoassay (LFIA) is a paper-based platform with extensive application in point-of-care (POC) testing and many fields. However, its clinical application is severely limited due to the lack of quantitative ability of standard LFIA tests; this augmentation provides the system with quantifying the signal from magenta-colored AuNPs. To address this issue, we proposed an ultra-compact optical system that allowed LFIAs to be performed more accurately and objectively. The experimental setup consisted of multiple optical accessories manufactured by 3D printing (STEP files were included). A high-resolution printer was used to print out a magenta card model for the LFIA, whose color code, ranging from 255, 255, 255 to 255, 0, 255 in the RGB (red, green, blue) format, represents different levels of magenta color intensity (from 0% to 100%) and thus the results of LFIA test strips. A mathematical model was built using a calibration curve to describe the relationship between magenta color value and reflectance spectrum. In addition, a spectrum module was integrated into the proposed system to identify and quantify LFIA results. This integration represents a pioneering step in developing portable detection techniques that facilitate quantifying LFIA results. Finally, we expect this ultra-compact optical spectroscopy system to have great potential for novel clinical applications.

## 1 Introduction

A lateral flow immunoassay (LFIA) is an easy-to-use, low-cost, and rapid test. These strengths make it one of the most widely used methods for point-of-care testing (POCT) [1, 2, 3]. When the LFIA—which uses a multi-layered test strip—is conducted, the sample is dripped onto the sample pad, then absorbed at a reasonable rate by the absorbance pad at the end of the test strip from the conjugate pad, which contains a detected antibody modified with gold nanoparticles (AuNPs) spread in advance [4, 5]; the sample, while flowing toward the end of the strip, passes through the test line, which is scattered with captured antibodies and whose color intensity of the test line varies depending on the concentration of the target analyte tested [6, 7].

The LFIA is widely adopted in the prevention of contagious diseases and plays a critical role in research into the identification of infectious sources [7, 8, 9]. This method treats the target as an antibody. When the host is infected with a virus, the immune system responds by producing antibodies (or immunoglobulins); in this case, a viable screening method can be employed where the concentration of immunoglobulins in the host's blood is measured through the LFIA biosensor. However, it should be noted that the LFIA rapids test only yields positive or negative results by performing qualitative analysis. On the other hand, spectral measurement techniques perform a quantitative antibody test. The concentration of immunoglobulins in the blood can be measured to estimate infection duration and determine the probability of a cytokine storm [10, 11]. Therefore, a biomedical spectrometer can be integrated as a reader to improve the reliability of the LIFA screening results [12, 13, 14].

Previous studies have shown that micro-sized AuNPs produce reds from light to dark that are visible to the naked eye when they react with the aggregation of gold nanoparticles. This color reaction is related to the size and shape of gold nanoparticles [15, 16]. Therefore, if the rapid test strips used for the LFIA react with a particular disease or symptom, their test lines turn magenta, whose concentration indicates the mild, moderate, or severe level of the condition examined [4, 17]. The naked eye determines the results obtained from LFIA rapid test strips, but they are subjectively interpreted. For a more precise interpretation of the results, one needs medical testing devices used in the healthcare system. However, such devices are costly; it is not easy to develop a low-cost but accurate testing device [18, 19, 20]. Combining with optoelectronic technology, colorimetric seems a good start in respect of detection for the POCT [21, 22, 23]. Nowadays, more optical sensors adopt RGB values to quantify the results. However, continuous spectral signals provide more information than RGB values [24]. For this reason, we proposed an ultra-compact optical spectroscopy system capable of conducting tests conventionally performed by medical-grade testing devices [12, 25, 26, 27].

In the present study, a high-resolution printer was used to print out a magenta card model for the LFIA whose color code, ranging from 255, 255, 255 to 255, 0, 255 in the RGB (red, green, blue) format, represents different levels of magenta color intensity (from 0% to 100%) and thus the results of LFIA test strips. Our ultra-compact optical spectroscopy system was produced from a 3D printer; it carried a micro spectral chip that harnessed sophisticated light traces to estimate magnified detection signals and thus measure spectral results in real time. Subsequently, a mathematical scale was developed using the raw data derived from the spectra, and the scale was adopted to quantify the experimental results. This study presented a low-cost and accurate testing ultra-compact optical spectroscopy system that came with LFIA test strips that are widely used. The plan was expected to set standards for the quantification of LFIA results. Our proposed testing system can significantly reduce misinterpretations of LFIA results. It can be used to conduct an LFIA without having any expert around, thereby minimizing the waste of healthcare resources and making it easier for researchers in academia and industry to perform LFIA-related experiments [28, 29, 30].

## 2 Material and method

### 2.1 Preparation of magenta color sample by a high-resolution printer

**Figure 1:**
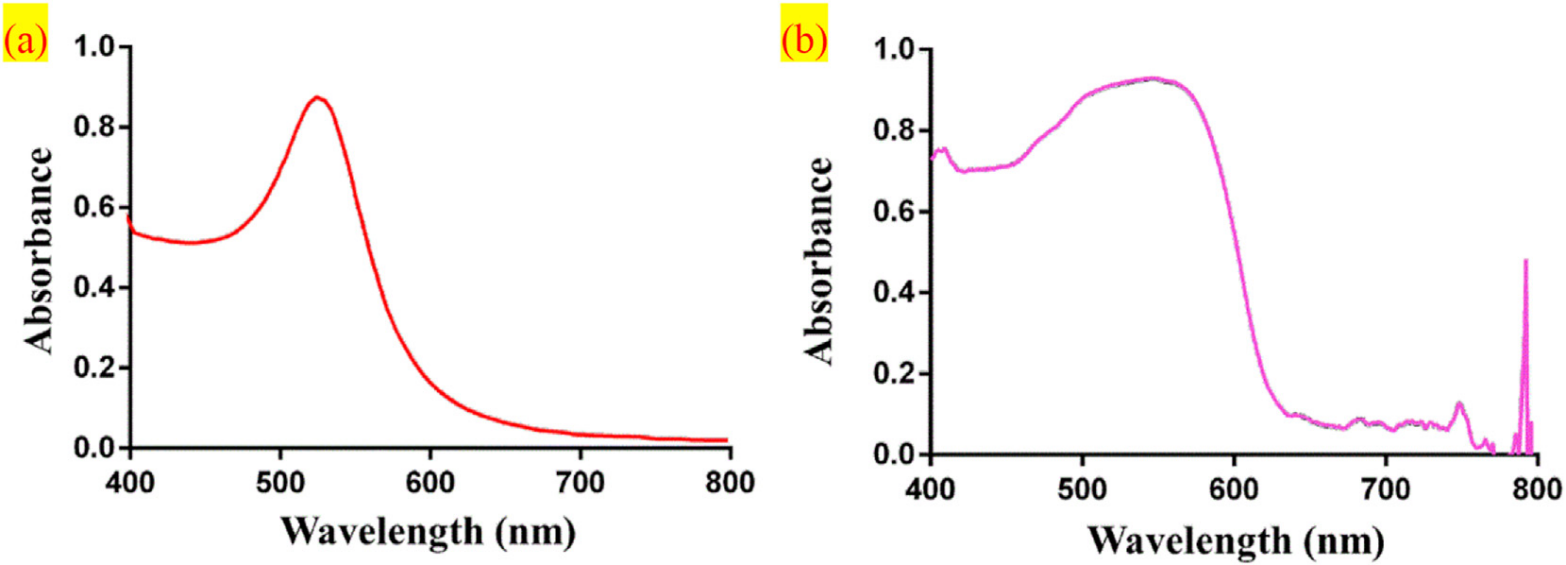
(a) AuNPs absorption spectrum versus (b) Magenta color absorption spectrum.

However, preparing samples for reaction during an LFIA is a highly challenging biochemical experiment; they are usually prepared in a lab with a biosafety level of 2 or higher and by biochemistry specialists. Past studies have suggested that LFIA signals are characterized by micro-sized AuNPs, which produce reds from light to dark that are visible to the naked eye when the aggregation of gold nanoparticles, color reaction is related to the size and shape of gold nanoparticles [4, 5, 17]. In this study, we compared the respective absorption spectra of AuNPs and magenta samples for the LFIA after the particles underwent reaction and found that both spectra shared highly similar characteristics (550–600nm) (Figure 1). Therefore, an optical spectroscopy system was developed to measure a series of signals from the absorption spectra of the magenta samples, with a database created for the examination of actual AuNP samples.

**Figure 2:**
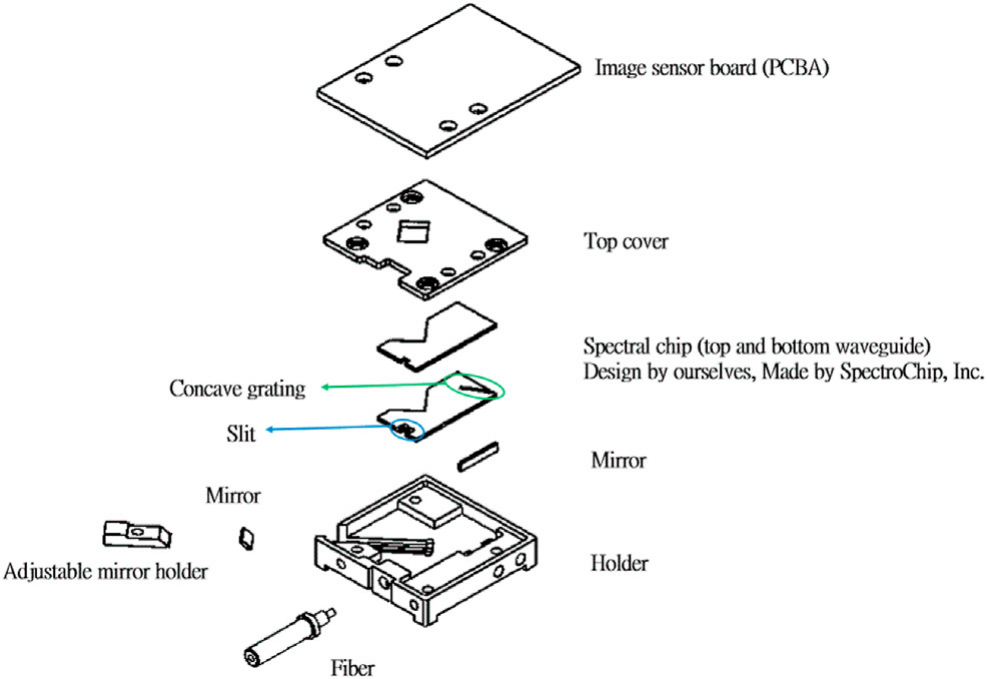
Exploded diagram of the micro spectral module.

During the experiment, 11 magenta samples with different levels of color intensity were printed out using a high-resolution printer and attached to 7 cm × 2 cm acrylic boards (Supplementary Appendix: S-Fig. 1). A self-developed optical recognition system was used to measure the reflectance spectra of the cards, and a mathematical model was then constructed to describe the relationships between spectral reflectance and magenta color intensity. The LFIA results were represented by the color intensity of the samples, which varied from as high as 100% (R, G, B = 255, 0, 255) to as low as 0% (R, G, B = 255, 255, 255,) (Supplementary Appendix: S-Fig. 2).

### 2.2 Optic experimental setup for reflectance spectrum measures

This research demonstrated a portable optical spectroscopy system with high precision, and low-cost was demonstrated. Figure 2 shows the exploded diagram of the micro spectral module. The grating structure of a micro spectral chip is designed and produced by micro-electro-mechanical systems (MEMS).

**Figure 3:**
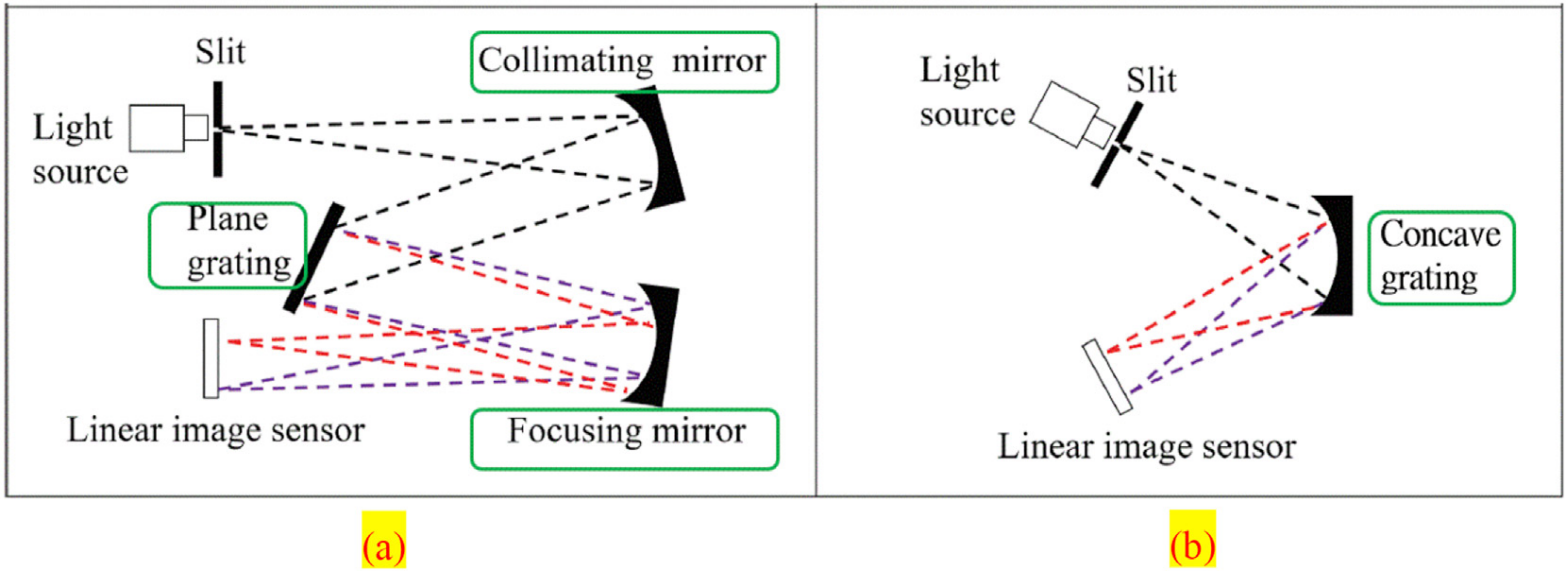
Comparison of the different light paths in the (a) plane grating and (b) concave grating.

The conventional plane grating spectrometer is bulky, complex, and expensive and is composed of sophisticated optical components (i.e., a slit, collimating mirror, plane grating, focusing mirror, and image sensor). We developed a spectral chip that used concave grating, which splits and focuses light simultaneously, simplifying light paths. Our chip consisted of the following optical components: a slit, concave grating, and image sensor. Thus, the chip could make a spectrometer smaller and lighter. Figure 3 describes the respective mechanisms underlying the operation of the conventional plane grating spectrometer's chip and our proposed spectral chip. MEMS are widely used in the design of optical components; the MEMS that can make a spectrometer smaller and reduce its production costs while allowing the device to maintain the same resolutions and photon conversion efficiency are collectively known as the micro-grating process. Our proposed spectral chip had a detectable wavelength range of 400–700 nm, a spectral resolution of 5–8 nm, and an entrance slit of 20 μm. The chip was assembled with an image sensor to yield a spectral module that was almost the size of a coin. Moreover, the chip was made of SU-8 thick-film photoresists and had a thickness of 125 μm Table 1 presents the specs of the spectral chip.

**Table 1:** Spectral chip specifications.

Parameters	Value
Spectral Resolution	5–8 nm
Wavelength Range	400–700 nm,
Entrance Slit	20μm
Structure of Grating	concave grating
Diffraction Order(m)	1
Grating pitch(d)	3μm
Cylindrical Height	125μm
Incident Arm Length(r)	15mm
Module Size	20 × 10.5 × 10 mm
ADC Bits	12
Number of Pixel Size	1280p

**Figure 4:**
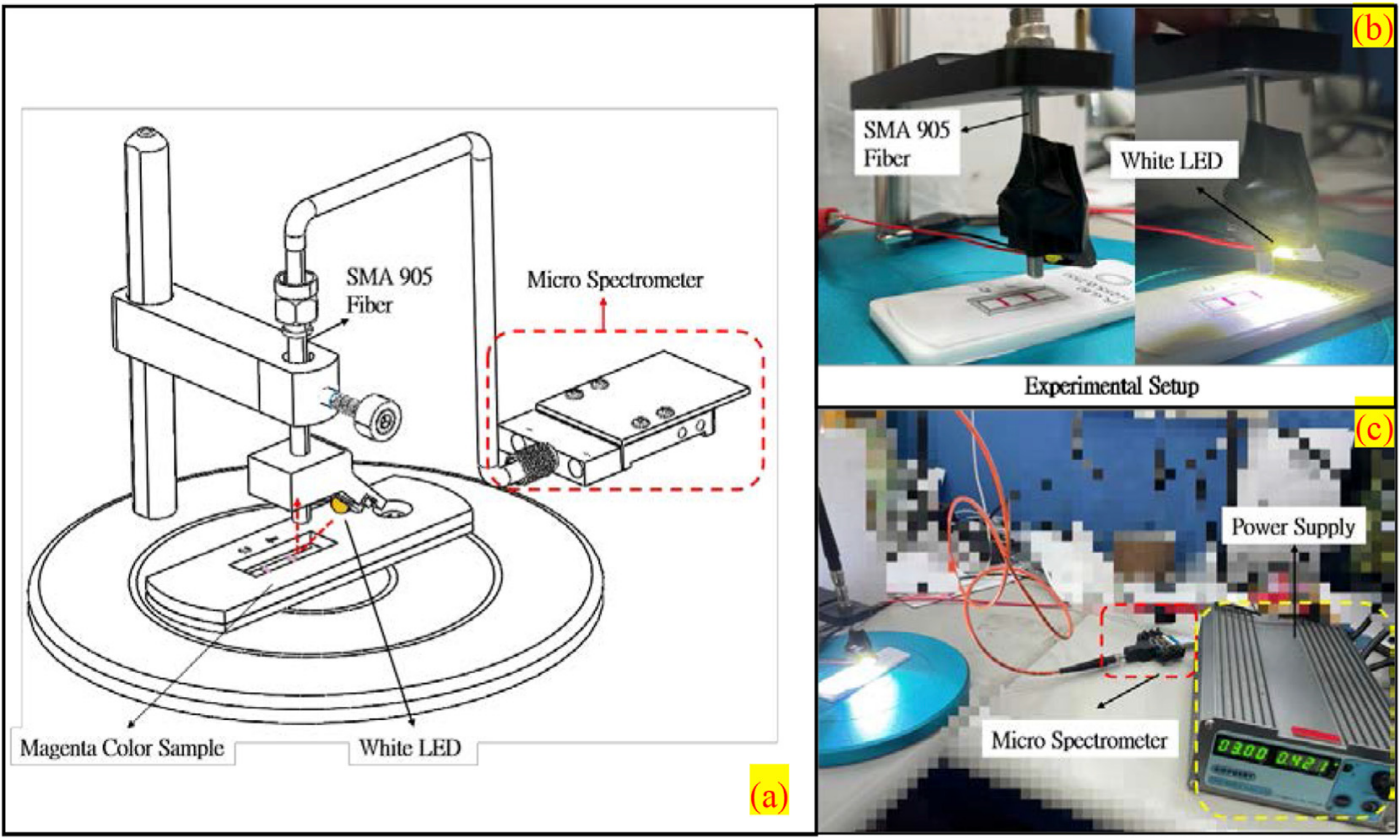
Experimental setup used to measure magenta color samples for spectrum (a) schematic diagram of pre-experimental design (b) experimental setup for measuring magenta samples and (c) turn on LED by power supplier.

The magenta test strips prepared for the LFIA—which represented different levels of concentration of AuNPs—varied from dark to light, or from 100% (R, G, B = 255, 0, 255) to 0% (R, G, B = 255, 255, 255), and were placed on a test platform. Expressly, an optical spectroscopy system was set up, above which a white LED light illuminated the test strips with different levels of magenta color intensity to create light reflections that were subsequently transferred via the standard SMA-905 optical fiber to the micro-spectrometer for analysis. The analysis was performed based on the reflectance spectra obtained from test strips with different levels of magenta color intensity so that a mathematical model could be created describing the relationship between magenta color intensity (%) and spectral reflectance. Using such a model, one may design a chart depicting how spectral reflectance changes at each magenta color intensity and apply the chart to more accurately determine any unknown magenta color intensity. The experiment was dispensed with preparing biochemical liquids and proved the feasibility of our proposed optical spectroscopy system. Figure 4 describes the setup and procedure of the experiment.

### 2.3 Theoretical background

Concave grating uses only one component to split and focus lights simultaneously. This can reduce the losses of light energy that occur during light reflection and make the light path a lot shorter and thus the entire spectrometer smaller. The spectral module, manufactured by SpectroChip, Inc., accurately determined the locations of emergent and incident light paths by using the equation for diffraction grating, as expressed by Eq. (1), where *m* is an integer that denotes the diffraction order, *λ* is the wavelength of the light source, *d* is the spacing between grating fringes, α is the angle of incidence, and *β* is the angle of emergence [31, 32, 33].

(1) $$\sin\;\alpha +\sin\;\beta =\frac{m\lambda }{d}$$

The incident light is a laser beam that has converged. Once split by a grate, it spreads out at two different wavelengths within a space, in which case there appears the angle *δβ*, as expressed by Eq. (2). Moreover, before an experiment is set up, *δβ* can be estimated to predict the location of the emergent light at different wavelengths, from which spectral data can be obtained through a CMOS detector.

(2) $$\cos\;\beta \delta \beta =\frac{m}{d}\delta \lambda$$

### 2.4 Beer-Lambert law

If a ray of monochromatic light strikes the surface of a medium with some thickness and passes through the medium, part of the light's energy that is reflected is defined as reflection (R). On the other hand, if an amount of that energy is absorbed while passing through the medium, it is referred to as absorption (A). Moreover, if the light penetrates the medium and emanates, this phenomenon is defined as transmittance (T). Taking the earliest Kubelka-Munk equation into account, the ideal law of conservation of energy can be expressed by Eq. (3) [34, 35, 36]:

(3) A+R+T=1;

Under the law of conservation of energy, a light source that strikes a medium's surface and has some of its energy absorbed by the medium, the intensity of transmitted or reflected light will decrease. If the medium cannot transmit the light but reflect part of the light's energy, then the medium may have absorbed some light energy. The application of spectroscopy in examining a substance is expanded based on such a principle. Therefore, a simple mathematical relationship exists between absorbance and the concentration of light-absorbing substances in a sample, and this relation is defined as the Beer-Lambert law, which is expressed by Eq. (4), where *A* is a measure of absorbance, $I_{0}$ is the incident light, $I_{t}$ Is the transmitted light, *T* is a measure of transmittance, *ε* is the extinction coefficient or molar absorptivity, *b* is the light-path length (cm), and c is the molar concentration of the sample [37, 38, 39].

(4) $$A=\log\frac{I_{0}}{I_{t}}=\log\frac{1}{T}=\varepsilon bc$$

The magenta samples prepared for this study exhibited changes in their reflectance and absorbance spectra. Therefore, the color intensity of these samples can be accurately estimated, considering the differences in both spectra. Employing such a procedure, a given spectral wavelength was selected based on how the concentration of the samples changed at different wavelengths. The absorbance (A) of the spectra of the samples was measured under similar environmental conditions; the selected characteristic wavelength was substituted into a relation equation for the absorption spectrum and magenta color intensity to determine the magenta color intensity of these samples. This way, a mathematical model describing the relationship between spectral absorbance and sample concentration for the spectral wavelength were constructed, and this model was defined as a calibration curve. After creating a good calibration curve, magenta samples of unknown color intensity were examined using the spectrometer.

### 2.5 Kubelka-Munk theory

According to the K-M theory, a medium can take advantage of reflectance spectroscopy to exhibit its optical properties, of which absorption and scattering can be considered when the concentration of a substance is quantified. This theory posits spectral differences in the absorption or distribution of a medium, depending on the concentration and content of a substance. Generally, light transmitted through a turbid medium is absorbed and scattered; the intensity of the light decreases, and backscattering occurs. Derived from the K-M theory, the most widely used equation for spectral measurement can be expressed by Eq. (5), where a0 and r0 are, respectively, absorption and reflection [40, 41, 42, 43].

(5) $$F\left(R_{\infty }\right)=\frac{{\left(1-R_{\infty }\right)}^{2}}{2R_{\infty }}=\frac{a0}{r0}$$

Absorption and scattering coefficients are defined as *K* and *S*, respectively, replacing those mentioned above a0 and r0. Both coefficients are assumed to have additivity *I* and concentration *ci*, as expressed by Eq. (6). Through this equation, the absorbance function for spectral measurement results can be determined to quantify spectral differences in relation to magenta color intensity [44, 45, 46].

(6) $$\frac{{\left(1-R_{\infty }\right)}^{2}}{2R_{\infty }}=\frac{a0}{r0}=\frac{K}{S}=\frac{\Sigma \left(ciKi\right)}{\Sigma \left(ciSi\right)}\approx \frac{\Sigma \left(ciKi\right)}{S}$$

### 2.6 Data fitting spectral signal-to-noise ratio optimization: Savitzky-Golay algorithm

The Savitzky–Golay smoothing filter, or the S–G filter, is widely used to smooth signals. It performs polynomial curve fitting on data points within a moving window (the filter shifts to the left and right for *m* points from the top middle position of the window), using the least squares method to yield a fitting equation that replaces the data at the center of the window. Such a filtering procedure minimizes raw data distortion and reduces noise interference. The equation for the S–G filter assumes $Y=\left[\begin{array}{ccccccc} y_{-m} & y_{-\left(m-1\right)} & \cdots & y_{0} & \cdots & y_{m-1} & y_{m} \end{array}\right]$ and treats the *i*-th element value as $y_{i}\;\left(i=-m\;,\ldots ,0,\ldots ,m\right)$, which consists of 2 m + 1 value. This dataset is fitted by the *n*-th polynomial [47, 48, 49]. The polynomial function for providing the data is expressed by Eq. (7):

(7) $$f\left(i\right)=\sum_{k=0}^{n}c_{k}i^{k}=c_{0}+c_{1}i+\ldots +c_{n}i^{n}$$

Then the error sum of squares (E) is estimated using Eq. (8):

(8) $$E=\sum_{i=-m}^{m}{\left(f\left(i\right)-y_{i}\right)}^{2}=\sum_{i=-m}^{m}{\left(\sum_{k=0}^{n}c_{k}i^{k}-y_{i}\right)}^{2}$$

The least squares method is used to find a set of coefficients **C** = [*c*0*c*1⋯*cn*] T so that the minimal error sum of squares (E) can be obtained. Thus, a partial derivative of 0 is performed on the coefficient *cr* (*r* = 0, 1, …,n) of the error sum of squares (E), as expressed by Eq. (9):

(9) $$\frac{\partial E}{\partial c_{r}}=0=\frac{\partial }{\partial c_{r}}\left\{\sum_{i=-m}^{m}{\left(\sum_{k=0}^{n}c_{k}i^{k}-y_{i}\right)}^{2}\right\}=2\sum_{i=-m}^{m}\left(\sum_{k=0}^{n}c_{k}i^{k}-y_{i}\right)i^{r}\;,r=0,1,2,\ldots \;,n$$

After the terms of Eq. (9) are reordered, Eq. (10) is obtained:

(10) $$\sum_{k=0}^{n}\left(\sum_{i=-m}^{m}c_{k}i^{k+r}\right)=\sum_{i=-m}^{m}y_{i}i^{r}\;,r=0,1,2\;,\ldots ,n$$

Thus, there are *n* + 1 equations for solving *n* + 1 variables from $c_{0}$ to $c_{n}$. To efficiently obtain fitting coefficients, the equation above is rewritten as matrix equation A, which is expressed by Eq. (11):

(11) $$A={\left[\begin{array}{ccccccc} {\left(-m\right)}^{0} & \cdots & {\left(-1\right)}^{0} & 1 & 1^{0} & \cdots & m^{0}\\ {\left(-m\right)}^{1} & \cdots & {\left(-1\right)}^{1} & 0 & 1^{1} & \cdots & m^{1}\\ \vdots & \vdots & \vdots & \vdots & \vdots & \vdots & \vdots \\ {\left(-m\right)}^{n} & \cdots & {\left(-1\right)}^{n} & 0 & 1^{n} & \cdots & m^{n} \end{array}\right]}^{T}$$

In Eq. (10), the vector *y* and the coefficient vector *C* share a relation expressed by Eq. (12), and this equation is then simplified as the generation solution of the coefficient vector *C*:

(12) $$A^{T}AC=A^{T}y;\;C={\left(A^{T}A\right)}^{-1}A^{T}y=Hy\;,H={\left(A^{T}A\right)}^{-1}A^{T}$$

The value output by the S–G filter is the value of the window center—i.e., the value obtained when i=0 is substituted into the filter. In Eq. (7), i=0 is the coefficient $c_{0}$. Moreover, $c_{0}$ is the dot product of *y* with the 1st row of H in Eq. (12). Thus, the relation between the input and output of the S–G filter is expressed by Eq. (13):

(13) $$c_{0}=h_{1}y_{-m}+h_{2}y_{-\left(m-1\right)}+\ldots +h_{2m+1}y_{m}$$

**Figure 5:**
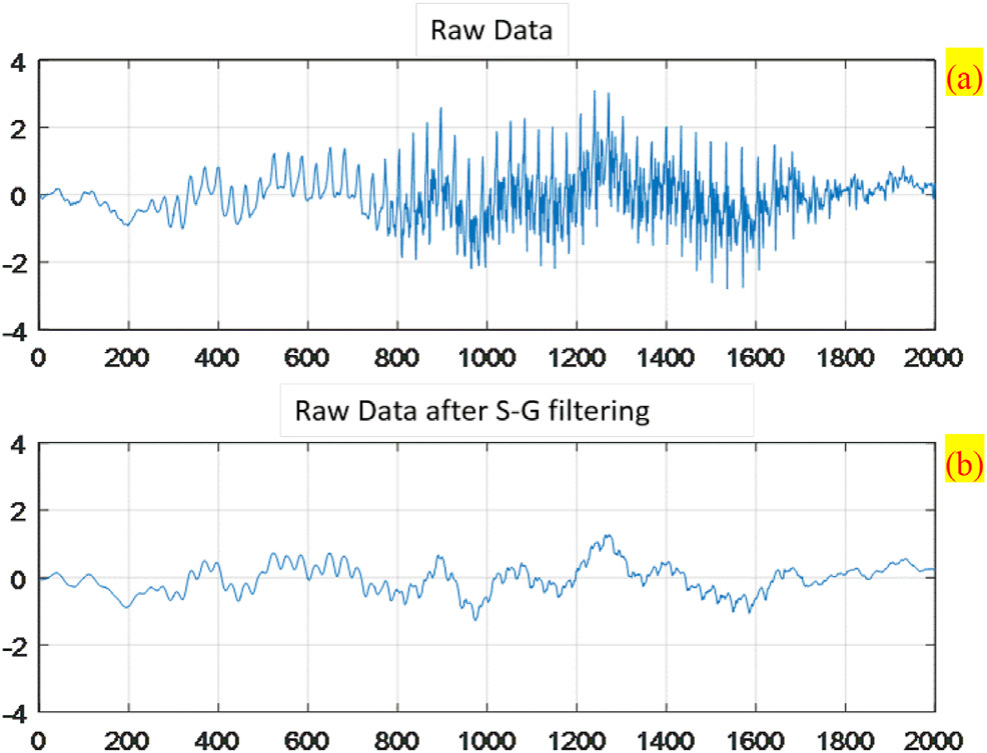
Data comparison (a) raw data without Savitzky-Golay algorithm (b) Raw data processing after Savitzky-Golay algorithm.

After the relation is simplified, the public function library y = sgolayfilt (x, order, frame length) can be found in MATLAB. This function library, which pairs the raw data with a polynomial order and frame length to be processed, performs S–G filtering on the data to yield smoothed data. Figure 5 compares the raw data before and after S–G filtering (The *X* and *Y* in this figure are the dimensionless row and column, respectively.)

### 2.7 Data analysis

In the experiment, the reflectance spectrum ($I_{magenta\left(n\right)\%}$) of the magenta samples, the reflectance spectrum ($I_{standard}$) of the white region on the samples, as well as a dark spectrum $\left(I_{dark}\right)$ when the lights were turned off, were measured respectively [49]. The reflectance of the samples, which differed in magenta color intensity, was defined as $R_{magenta\left(n\right)\%}$ and estimated using Eq. (14):

(14) $$R_{magenta\left(n\right)\%}=\frac{I_{magenta\left(n\right)\%}-I_{dark}}{I_{standard}-I_{dark}}$$

Based on Eq. (4), the measurement of the absorption spectrum $A_{magenta\left(n\right)\%}$ As is expressed by Eq. (15):

(15) $$A_{magenta\left(n\right)\%}=-\log\frac{I_{magenta\left(n\right)\%}-I_{dark}}{I_{standard}-I_{dark}}$$

**Figure 6:**
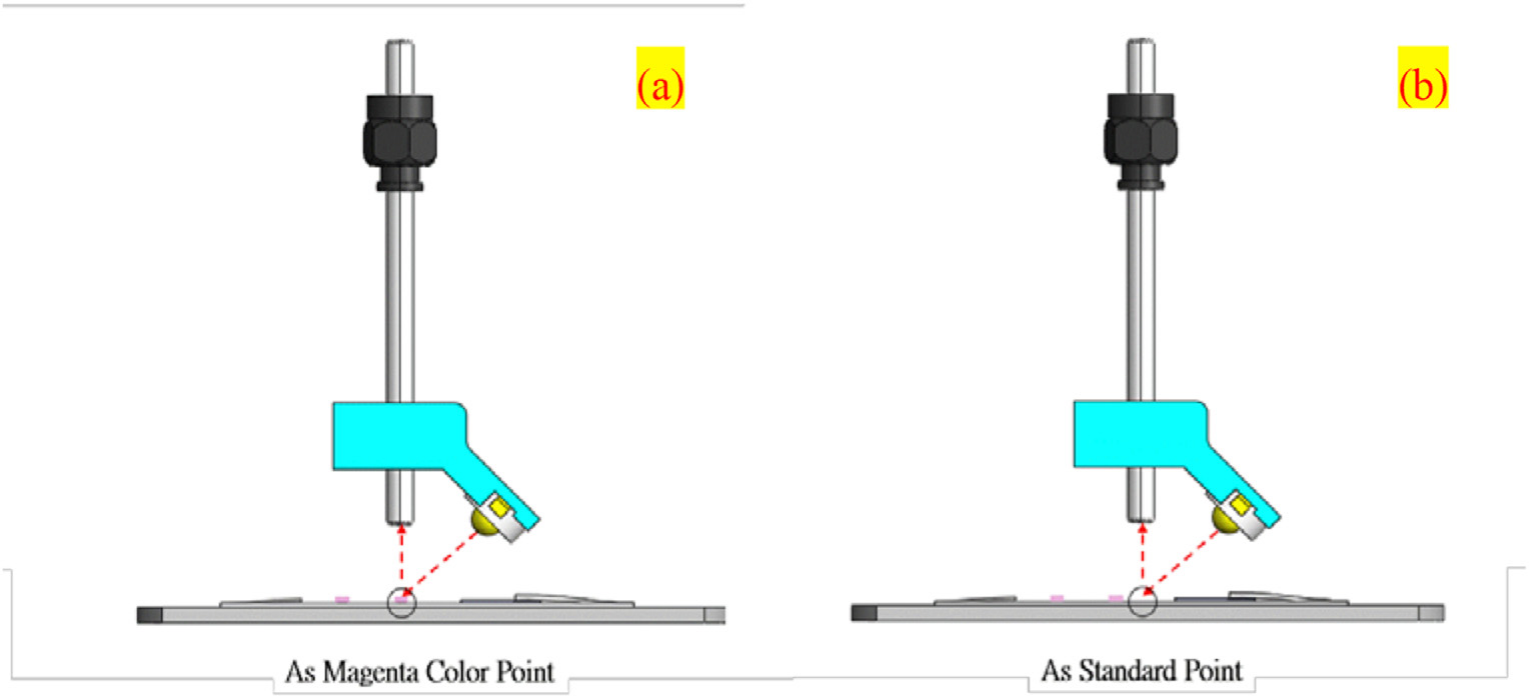
(a) Indicate the detection position of the magenta color region and (b) the standard white region.

A calibration curve was created for different levels of magenta color intensity, which were shown in the reflectance spectrum ($I_{magenta\left(n\right)\%}$). All the levels of magenta color intensity were statistically quantified in numerical vs. percentage terms. The white and magenta regions examined are respectively illustrated in Figure 6.

### 2.8 Hill equation

The Hill equation describes the score of the saturation of macromolecules by ligands as a function of ligand concentration. The equation determines the degree of cooperativity of the ligands binding to the enzyme or receptor. In this equation, the Hill coefficient (h) measures the cooperativity between ligands and the macromolecules they have saturated. The equation was used to analyze the experiment results [50].

### 2.9 Magenta color level versus reflectance spectroscopy for quantitation

Within the setup of the experiment introduced earlier, the dark spectrum $I_{dark}$ Obtained when the lights were off was first measured and defined as a spectrum without any sample in place or incident light provided. Next, the reflectance spectra of the pieces were estimated, taking into account the levels of magenta color intensity from 100% to 0%: $I_{magenta\left(100\right)\%}$, $I_{magenta\left(90\right)\%}$, $I_{magenta\left(80\right)\%}$, $I_{magenta\left(70\right)\%}$, $I_{magenta\left(60\right)\%}$, $I_{magenta\left(50\right)\%}$, $I_{magenta\left(40\right)\%}$, $I_{magenta\left(30\right)\%}$, $I_{magenta\left(20\right)\%}$, $I_{magenta\left(5\right)\%}$, and $I_{magenta\left(0\right)\%}$. The white region on the samples was used as the reference sample ($I_{magenta\left(0\right)\%}$), whose reflectance spectrum ($I_{standard}$) was measured. The raw data were converted into $R_{magenta\left(n\right)\%}$ and $A_{magenta\left(n\right)\%}$ using Eqs. (14) and (15), respectively. In addition, the reflectance spectra of the samples were found to have higher resolutions at a wavelength (*λ*) of 575 nm, where they exhibited more noticeable differences in magenta color intensity. Thus, calibration curves were created with the reflectance spectra at *λ* = 575 nm.

## 3 Result

In the experiment, a reflectance spectrum with lights off was obtained and used as a dark spectrum $\left(I_{dark}\right)$ That was the sum of electrical and background noises without the presence of light. Next, the reflectance spectrum was acquired from the white region on the samples while the lights were on (Figure 6) and used as the reference spectrum ($I_{standard}$). Finally, the reflectance spectra of magenta samples $(I_{magenta\left(100\right)\%}$, $I_{magenta\left(90\right)\%}$, $I_{magenta\left(80\right)\%}$, $I_{magenta\left(70\right)\%}$, $I_{magenta\left(60\right)\%}$, $I_{magenta\left(50\right)\%}$, $I_{magenta\left(40\right)\%}$, $I_{magenta\left(30\right)\%}$, $I_{magenta\left(20\right)\%}$, $I_{magenta\left(5\right)\%}$, and $I_{magenta\left(0\right)\%})$ were respectively measured. All these signals—$I_{magenta\left(100∼0\right)\%}$—were derived by sampling the raw data three times, averaging the data, and substituting them into Eq. (14) to yield ${(R}_{magenta\left(100∼0\right)\%}$; $R_{magenta\left(0\right)\%}$, in particular, had an RGB value of 255, 255, 255. Therefore, $I_{standard}$ divided by $I_{magenta\left(0\right)\%}$ was ≈ 1. However, $R_{magenta\left(0\right)\%}$ was eliminated so that the measurement results of the raw data would comply with the law of conservation of energy, which is expressed by Eq. (3).

**Figure 7:**
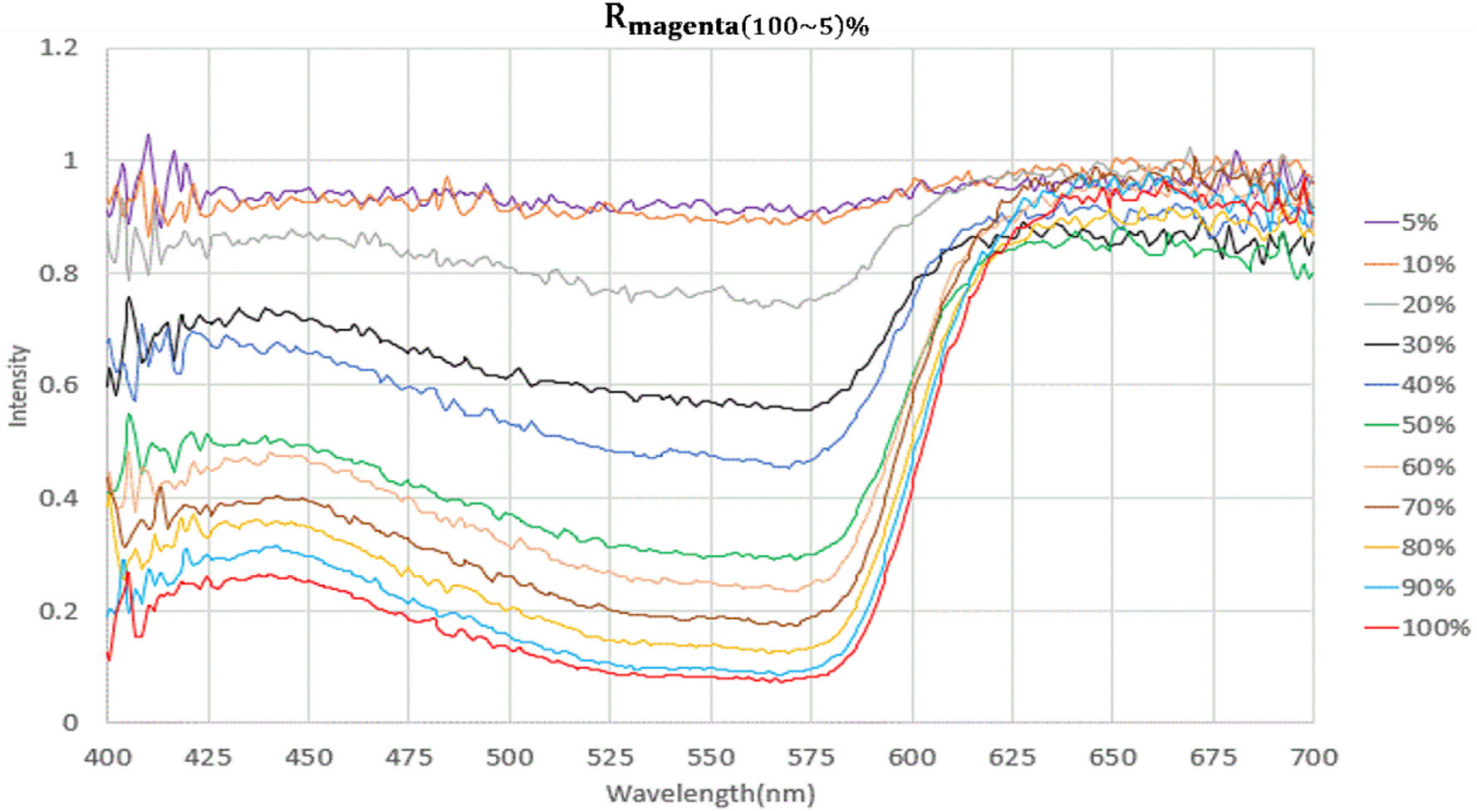
Reflectance spectra at different concentrations of magenta samples.

The experimental data were plotted to yield a graph of measurement results (Figure 7). As suggested by the figure, the darkest reflectance spectrum ${(R}_{magenta\left(100\right)\%}$) demonstrated more noticeable absorption but lower reflectance at a wavelength of 400–600 nm; the lightest one ${(R}_{magenta\left(5\right)\%}$) showed higher reflectance; and magenta color intensity exhibited marked and regular changes as the reflectance spectrum $(R_{magenta}$) varied. All these indicated that the experimental design and measurement results of this study were reliable.

**Figure 8:**
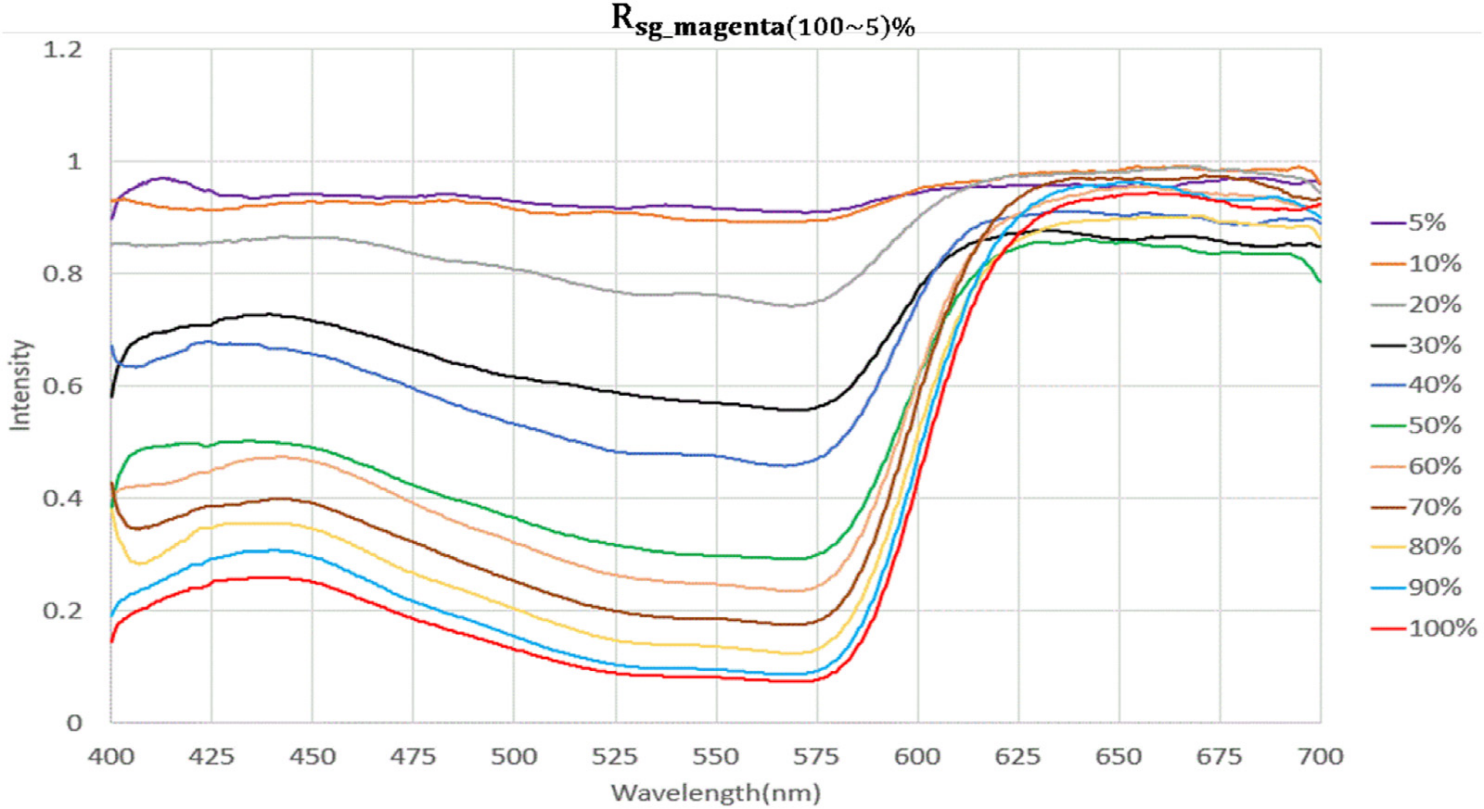
Reflectance spectra after Savitzky-Golay algorithm at different concentrations of magenta samples.

Figure 7 revealed that even after the raw data were sampled three times and averaged, many discrete points in the data were affected by signals with low magenta color intensity of $R_{magenta\left(5\right)\%}$, in which case the color intensity exceeded 1. Thus, the smoothness of the waves on the figure needed improving. $R_{magenta\left(100∼5\right)\%}$ were substituted into the Savitzky–Golay algorithm, with the order defined as 3 and frame length as 25. This S–G filtering process yielded $R_{sg\_magenta\left(100∼5\right)\%}$, as summarized in Figure 8. S–G filtering smoothed the uneven areas on the waves without distorting so that the reflectance spectra demonstrated high discriminability in terms of magenta color intensity.

The reflectance spectra ${(R}_{sg\_magenta\left(100∼5\right)\%})$ showed higher discriminability at *λ* = 575 nm, where magenta color intensity exhibited more noticeable differences. The numerical values corresponding to all levels of magenta color intensity processed through S–G filtering are summarized in the supplementary appendix (Supplementary Appendix: S-Table 1). As the supplementary information, the values decreased from 0.910717965 (with 5% magenta color intensity) to 0.077050501 (with 100% magenta color intensity). Because of this gradual decrease, a mathematical model describing the relationship between magenta color intensity and spectral reflectance was created, and this model was defined as a calibration curve. Such a procedure allowed us to retrieve the calibration curve of any S–G filtered reflectance spectrum with unknown magenta color intensity ${(R}_{sg\_magenta\left(unknown\right)\%}$) in the subsequent experiments and estimate its magenta color intensity.

**Figure 9:**
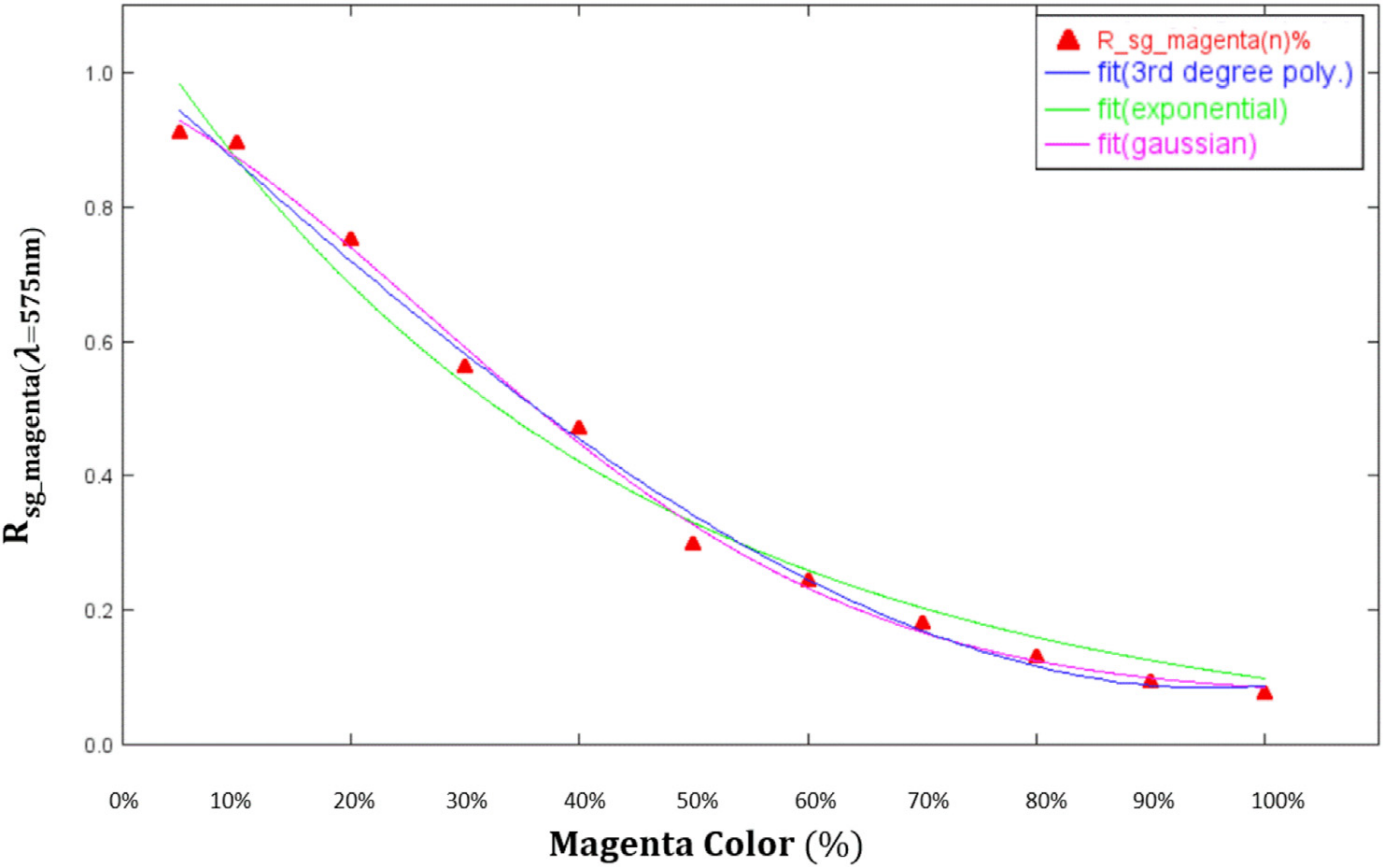
The fitting results were performed on Third-degree polynomial fitting, exponential fitting, and Gaussian fitting.

Third-degree polynomial, exponential, and Gaussian, respectively expressed by Eqs. 16, 17, and 18, were all performed on the numerical values shown in the supplementary appendix. The fitting results are shown in Figure 9. With *λ* = 575 nm, the Gaussian fitting results were the most similar to $R_{sg\_magenta\left(100∼5\right)\%}$. The value for the results—an R^2^ of 0.99662—represented a strong correlation between the spectral data and the fitting curve. This function was used as the calibration curve for the present study. The coefficients in Eq. (18) that applied to the experiment were reorganized to yield Eq. (19), where $R_{sg\_magenta\left(\lambda =575nm\right)}$ is a reflectance spectrum with *λ* = 575 nm when a sample measurement is performed and n (%) is the color concentration of magenta samples in the RGB format. This way, the calibration curve could be applied to determine the magenta color intensity of any $R_{sg\_magenta\left(\lambda =575nm\right)}$ with unknown magenta color intensity.

(16) $$y=a+bx+cx^{2}+dx^{3}$$

The obtained coefficients are as follows: a = 1.02048, b = -1.55461, c = 0.16517, d = 0.45659

(17) $$y=a*\exp^{bx}$$

The obtained coefficients are as follows: a = 1.10999, b = -2.42329

(18) $$y=a+\left(b-a\right)\exp^{\frac{{-\left(x-c\right)}^{2}}{2d^{2}}}$$

The obtained coefficients are as follows: a = 0.072335, b = 1.01360, c = -0.11643, d = 0.38106.

(19) $$R_{sg\_magenta\left(\lambda =575nm\right)}=0.072335+\left(0.941265\right)\exp^{\frac{{-\left(n\left(\%\right)+0.11643\right)}^{2}}{0.290413}}$$

**Figure 10:**
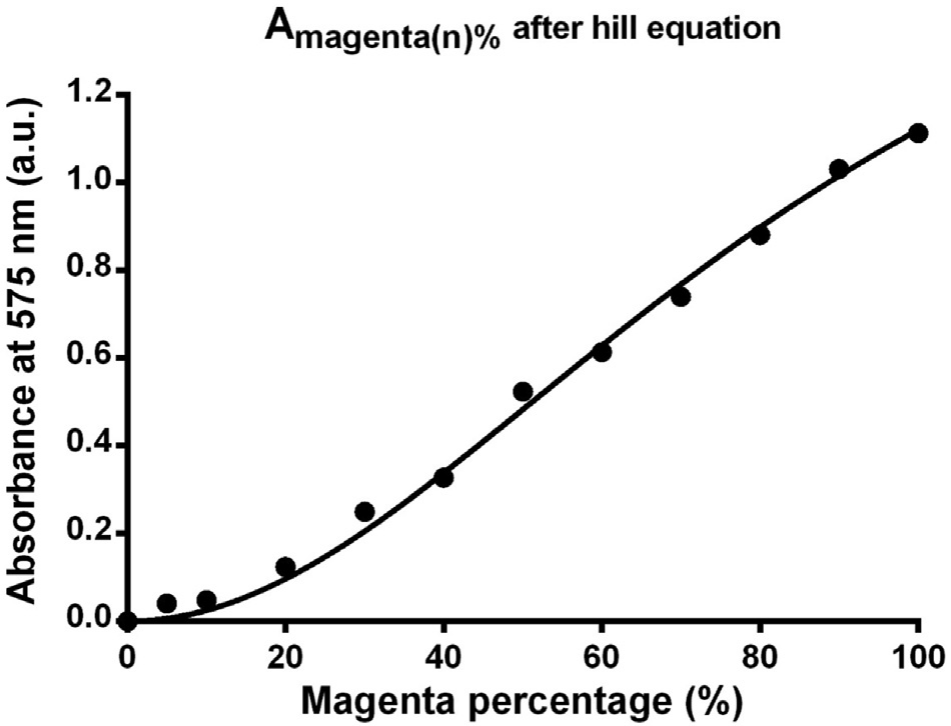
The fitting results obtained through the Hill equation.

The absorption spectra $A_{sg\_magenta\left(\lambda =575nm\right)}$ They were organized using Eq. (15) and summarized in the supplementary appendix (Supplementary Appendix: S-Table 2). The $A_{sg\_magenta\left(\lambda =575nm\right)}$ in supplementary information were subsequently input into the Hill equation, as expressed by Eq. (20). In this equation, $B_{\max}$ is the maximum specific binding in the same units as Y, X is $A_{sg\_magenta\left(\lambda =575nm\right)}$; $K_{d}$ is a dissociation constant that is widely used to describe whether the fitting of concentration (%) is excessively saturated; and h is the Hill coefficient (a dimensionless parameter), denotes the slope of the Hill equation, and describes the relationship between concentration and the equation. Figure 10 illustrates the fitting results obtained through the Hill equation. With $B_{\max}$ = 2, *h* = 2, $K_{d}$ = 88.62, and in particular an R^2^ of as high as 0.9956, the fitting curve indicated a strong correlation between magenta color intensity and absorbance. This fitting curve can be used when measuring actual biological samples.

(20) $$Y=B_{\max}\left(\frac{X^{h}}{{K_{d}}^{h}+X^{h}}\right)$$

## 4 Discussion

Techniques designed to identify infectious sources play a profound role in research into preventing and controlling infectious diseases. Numerous researchers have argued for the feasibility of using an LFIA biosensor to measure the concentration of immunoglobulins in the blood and employing a biomedical spectrometer as a reader to improve the reliability of the measurement results. Another benefit of using the spectrometer is that it filters sample noise [51, 52]. However, the spectrometer is a characteristically bulky medical device that is highly expensive and rarely accessible. Moreover, its large size, heavy weight, and low robustness make it too costly to use; for developing countries, such a device can cause a substantial financial burden. Even if an LFIA is performed, the specimen must be sent to a large healthcare institution for further analysis, but this may do little to help improve quarantine efficiency. The present study proposed a low-cost but accurate optical spectroscopy system with LFIA test strips applicable in different contexts and can be used to quantify LFIA measurement results. This system can significantly reduce misinterpretations of LFIA test results, and it can be used to conduct an LFIA without having any expert around, thereby minimizing the waste of healthcare resources and making it easier for researchers in academia and industry to perform LFIA-related experiments [2, 8, 12, 13, 30, 37].

This study found similarities between the absorption spectra of AuNPs and magenta samples. We used the spectra for magenta samples to develop a set of standards and showed that our proposed optical spectroscopy system can perform quantification. Thus, the color concentration of magenta samples can be evaluated as a simulated measurement process for a serum test.

In this study, a micro-spectrometer practically the coin size was used to develop an optical spectroscopy system. This system obtained the reflectance spectra of magenta samples illuminated by a white LED source; the spectral signals were sent to the spectrometer for analysis via the standard SMA-905 optical fiber with a diameter of 1 mm. The specs of this spectrometer are comparable to its equivalents commonly used in medical labs. Its chip is manufactured by the Taiwanese company SpectroChip. Inc., which leveraged remarkable semiconductor technologies to make optical components lighter and reduce costs. This production process is different from traditional spectrometers, consisting of a complex combination of optical components.

During the experiment, a high-precision printer was used to prepare samples with different levels of magenta color intensity ranging from 0% (R, G, B = 255, 255, 255) to 100% (R, G, B = 255, 0, 255). These samples were treated as the LFIA results and measured using the proposed optical spectroscopy system. The raw reflectance spectra of the samples were processed using the following equations: the S–G algorithm (which is included in the Beer-Lambert law and was used in this study to smooth absorption and reflectance spectra), fitting equations (Gaussian fitting, third-degree polynomial fitting, and exponential fitting), and the Hill equation (which determined whether the fitting of magenta color intensity was excessively saturated). Moreover, after Gaussian fitting was performed, strong correlations were found between different levels of magenta color intensity and reflectance spectra, suggesting that the spectral data and the fitting curve were closely related. This function was used as the calibration curve for the present study.

Although the results of this study seemed satisfactory, the study had the following limitations. First, the calibration curve for the magenta samples and the spectra for AuNPs require further experimental validation. Specifically, suppose a healthcare institution adopts our optical spectroscopy system. In that case, the institution should rigorously replicate the procedure of the experiment conducted in this study. However, doing so may lead to more data generated and less space for data storage. Second, we are still working on commercializing the proposed optical spectroscopy system and making it easier to use. The system is characteristically low-cost, and if it becomes ubiquitous, it certainly can be operated by a non-expert user. The system is also expected to be a reliable device for at-home point-of-care testing (POCT) [53, 54, 55, 56], allowing general users to track their health metrics.

## 5 Conclusion

Past studies in recent years have focused mainly on using LFIAs to examine analytes in clinical and non-clinical settings. While LFIAs offer multiple benefits (easy to perform, affordable, small in size, conducting analysis efficiently, and eliminating the need for experts) and have extensive application in clinical and non-clinical research, they have a limitation in that their test results are only deemed to be either positive or negative if examined by the naked eye. However, the color concentration of AuNPs after they have reacted can be quantified through a specialized device so that a medical condition can be either mild, moderate, or severe, depending on the concentration. We argued that LFIAs might lose their benefits if performed using conventional medical devices, which are expensive and cannot be operated without expert knowledge.

In this study, the proposed optical spectroscopy system was used to measure the reflectance spectra of magenta samples, a calibration curve was created at a wavelength of 575 nm, and the spectral data were processed through three fitting equations (Gaussian fitting, third-degree polynomial fitting, and exponential fitting)—with Gaussian fitting having yielded a strong correlation between the data and the fitting curve. This ultra-compact optical spectroscopy system is expected to be applicable in real LFIAs; by our conservative estimate, it may cost an affordable US$ 1 to 2 per test for one strip, and the optical spectroscopy system can be used multiple times for many tests. Particularly suitable for developing countries, home testing, and under-resourced communities. Moreover, it may become a reliable device for at-home POCT, allowing general users to track their health metrics.

## Declarations

### Author contribution statement

Wei-Huai Chiu: Conceived and designed the experiments; Performed the experiments; Analyzed and interpreted the data; Contributed reagents, materials, analysis tools or data; Wrote the paper.

Wei-Yi Kong: Analyzed and interpreted the data.

Yuan-Hui Chueh: Analyzed and interpreted the data.

Jyun-Wei Wen: Performed the experiments.

Ciao-Ming Tsai: Analyzed and interpreted the data.

Chitsung Hong: Contributed reagents, materials.

Pang-Yen Chen: Analyzed and interpreted the data.

Cheng-Hao Ko: Conceived and designed the experiments; Analyzed and interpreted the data; Wrote the paper.

### Funding statement

Cheng-Hao Ko was supported by 10.13039/100007225Ministry of Science and Technology, Taiwan [MOST 110-2218-E-011-008 & MOST 110-2218-E-011 -010 -MBK].

### Data availability statement

Data associated with this study has been deposited at https://scholar.google.com/citations?user=YRP4J7AAAAAJ&amp;hl=zh-TW.

### Declaration of interest’s statement

The authors declare no conflict of interest.

### Additional information

No additional information is available for this paper.

## Appendix A Supplementary data

$$$0930supplementary Appendix-V2 $$$0930supplementary Appendix-V2
