# Supplementary material for: Using an ultra-compact optical system to improve lateral flow immunoassay results quantitatively
Source: Heliyon. 2022 Dec 7;8(12):e12116. doi: 10.1016/j.heliyon.2022.e12116 (PMC9761723; doi:10.1016/j.heliyon.2022.e12116)
Supplement: $$$0930supplementary Appendix-V2 [file mmc1.docx]

**Supplementary Appendix**

Supplement to: Wei-Huai Chiu et al. Using an Ultra-Compact Optical System to Improve Lateral Flow Immunoassay Results Quantitatively.

SSRN: <https://ssrn.com/abstract=4170535>

DOI: [http://dx.doi.org/10.2139/ssrn.4170535](https://dx.doi.org/10.2139/ssrn.4170535)

The authors have provided this appendix to give readers additional information about the work.

S-Table 1 Reflectance spectra of magenta samples with different concentrations, lambda(𝜆) at 575nm

| Magenta Color (%) | $R_{\mathbf{sg}\_\mathbf{magenta}\left( \mathbf{n} \right)\%}$ , 𝜆=575 nm |
| --- | --- |
| 5% | 0.910717965 |
| 10% | 0.894909049 |
| 20% | 0.750417559 |
| 30% | 0.562045999 |
| 40% | 0.470538299 |
| 50% | 0.299472178 |
| 60% | 0.243229833 |
| 70% | 0.181824233 |
| 80% | 0.131515104 |
| 90% | 0.092999661 |
| 100% | 0.077050501 |

S-Table 2 Absorbance spectra of magenta samples with different concentrations, lambda(𝜆) at 575nm

| Magenta Color (%) | $A_{\mathbf{sg}\_\mathbf{magenta}\left( \mathbf{n} \right)\%}$ , 𝜆=575 nm |
| --- | --- |
| 5% | 0.040616 |
| 10% | 0.048221 |
| 20% | 0.124697 |
| 30% | 0.250228 |
| 40% | 0.327405 |
| 50% | 0.523644 |
| 60% | 0.613983 |
| 70% | 0.740348 |
| 80% | 0.881024 |
| 90% | 1.031519 |
| 100% | 1.113225 |


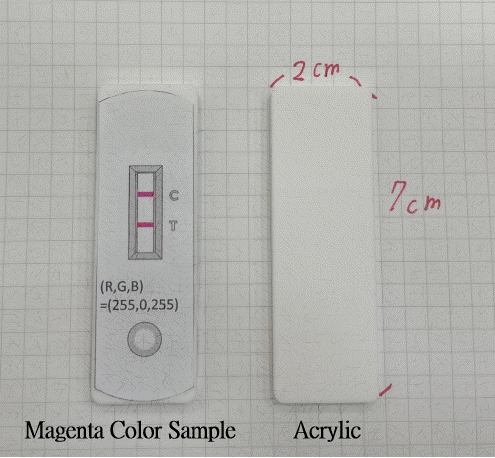


S-Fig 1. Color cards attached to 7 cm × 2 cm acrylic boards


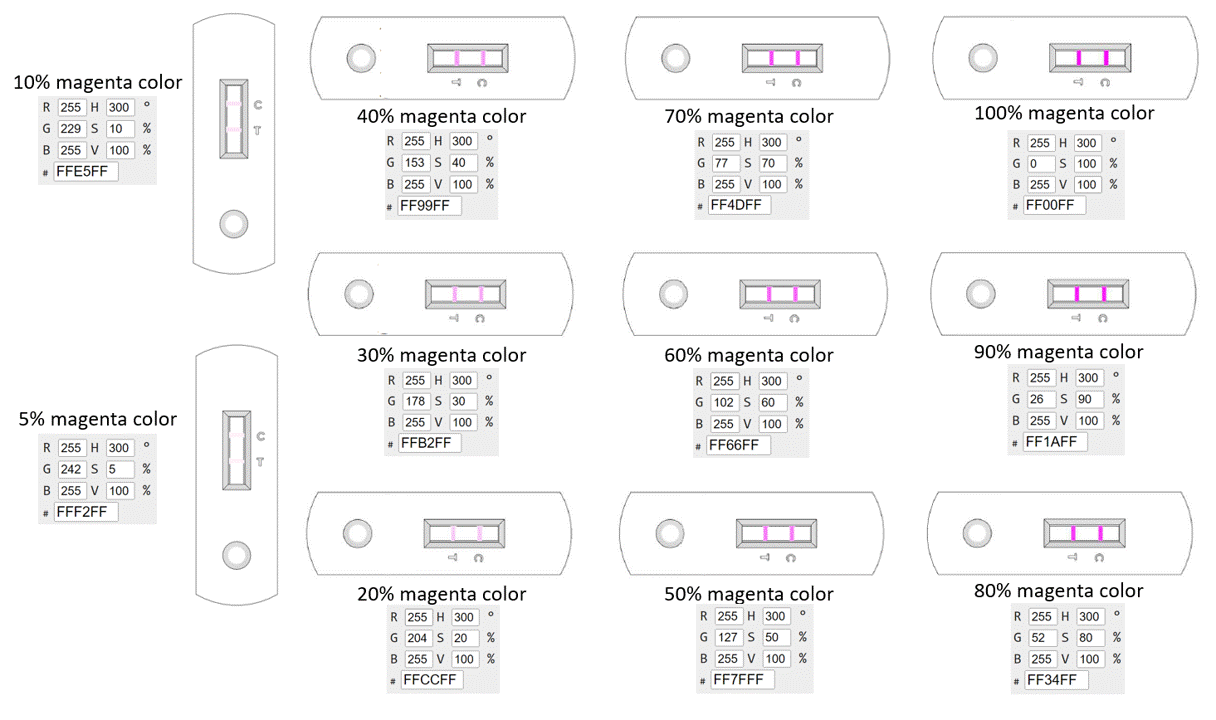


S-Fig 2. Magenta color samples as LFIA results from (R, G, B = 255, 0, 255,) to (R, G, B = 255, 255, 255,)
